# Cost-Effectiveness of a Specialized Oral Nutritional Supplementation for Malnourished Older Adult Patients in Spain

**DOI:** 10.3390/nu10020246

**Published:** 2018-02-22

**Authors:** María D. Ballesteros-Pomar, Diana Martínez Llinàs, Scott Goates, Rebeca Sanz Barriuso, Alejandro Sanz-Paris

**Affiliations:** 1 Servicio de Endocrinología y Nutrición, Complejo Asistencial Universitario de León, Altos de Nava, 24071 León, Spain; mdballesteros@telefonica.net; 2 Oblikue Consulting, Carrer del Comte d’Urgell 240 2ºD, 08036 Barcelona, Spain; diana.martinez@oblikue.com; 3 Health Economics and Outcomes Research, Abbott Nutrition R&D, Building ES1 East 2900 Easton Square Place, Columbus, OH 43219, USA; scott.goates@abbott.com; 4 Scientific Affairs & Training, Abbott Nutrition International, Abbott Laboratories, S.A., Avenida de Burgos 91, 28050 Madrid, Spain; 5 Servicio de Endocrinología y Nutrición, Hospital Universitario Miguel Servet, Paseo Isabel la Católica 1–3, 50009 Zaragoza, Spain; sanzparisalejandro@gmail.com; 6 Instituto de Investigación Sanitaria Aragón (IIS-Aragón), Hospital Universitario Miguel Servet, Paseo Isabel la Católica 1-3, 50009 Zaragoza, Spain

**Keywords:** malnutrition, oral nutritional supplement, cost-effectiveness, Spain, beta-hydroxy-beta-methylbutyrate, NOURISH study

## Abstract

Malnutrition has been related to prolonged hospital stays, and to increases in readmission and mortality rates. In the NOURISH (Nutrition effect On Unplanned Readmissions and Survival in Hospitalized patients) study, administering a high protein oral nutritional supplement (ONS) containing beta-hydroxy-beta-methylbutyrate (HP-HMB) to hospitalised older adult patients led to a significant improvement in survival compared with a placebo treatment. The aim of this study was to determine whether HP-HMB would be cost-effective in Spain. We performed a cost-effectiveness analysis from the perspective of the Spanish National Health System using time horizons of 90 days, 180 days, 1 year, 2 years, 5 years and lifetime. The difference in cost between patients treated with HP-HMB and placebo was €332.75. With the 90 days time horizon, the difference in life years gained (LYG) between both groups was 0.0096, resulting in an incremental cost-effectiveness ratio (ICER) of €34,700.62/LYG. With time horizons of 180 days, 1 year, 2 years, 5 years and lifetime, the respective ICERs were €13,711.68, €3377.96, €2253.32, €1127.34 and €563.84/LYG. This analysis suggests that administering HP-HMB to older adult patients admitted to Spanish hospitals during hospitalisation and after discharge could be a cost-effective intervention that would improve survival with a reduced marginal cost.

## 1. Introduction

Malnutrition is a common condition in hospitalised patients. Observational studies carried out in Spanish hospitals have estimated a prevalence of malnutrition of between 23% and 70% [1,2,3,4,5,6,7], depending on the population assessed and the screening and assessment tools used. Advanced age is a known risk factor for malnutrition [4].

Malnutrition has been related to an increased risk of postoperative infections and complications, extended stays in hospital and an increase in readmissions and mortality rates [8,9,10,11,12]. This is accompanied by an increase in costs. It has been estimated that, in Spain, on average, malnutrition prolongs hospital stay by three days, with an overall annual cost of at least €1143 billion [13].

In systematic reviews, the use of high protein oral nutritional supplements (ONS) has been linked to clinical, nutritional and functional improvements and significant reductions in the number of readmissions [14,15,16]. More specifically, using high protein ONS with beta-hydroxy-beta-methylbutyrate (HP-HMB) has been associated with improvements in muscle mass and function in adults both in hospital and community environments [16]. However, evidence on the clinical benefits of nutritional supplements on mortality is limited [17].

In the Nutrition effect On Unplanned Readmissions and Survival in Hospitalized patients (NOURISH) study, the use of HP-HMB showed a decrease, compared with a placebo treatment, in mortality risk in the 90-day post-discharge period (4.8% vs. 9.7%; relative risk (RR): 0.49; *p* = 0.018) in older adults (≥65 years of age) admitted to hospital for congestive heart failure (CHF), acute myocardial infarction (AMI), pneumonia or chronic obstructive pulmonary disease (COPD) [18]. In the NOURISH study, all patients received standard nutritional care, regardless of whether they were in the intervention or control group. Administering HP-HMB also proved to be a cost-effective intervention in a study carried out in the USA, where incremental cost-effectiveness ratios (ICER) were of US $33,818 per quality-adjusted life year (QALY) and $524 per year of life year gained (LYG), respectively, using 90-day and lifetime time horizons [19].

The aim of this study is to determine whether administering HP-HMB would be cost-effective in patients admitted to Spanish hospitals, using short, medium and long-term time horizons, applying the data from the NOURISH study to the Spanish population.

## 2. Patients and Methods

Costs and effects of the nutritional intervention were assessed in a hypothetical cohort of patients with the baseline characteristics of the patients included in the NOURISH study [18]. The mean age of the patients included in the NOURISH study was 78.1 in the control group and 77.7 in the HP-HMB group. In the placebo group, 86.7% of patients were mild–moderately malnourished (SGA B) and 13.3% were severely malnourished (SGA C); whereas, in the HP-HMB group, the respective rates for mild–moderate and severe malnourishment were 87.9% and 12.1%, respectively. The most frequent primary diagnosis was COPD (34.1% in the control group and 34.8% in the HP-HMB group), followed by pneumonia (32.5% and 30.4%), CHF (25.3% and 25.2%) and AMI (8.1% and 9.6%) [18].

We carried out an incremental cost-effectiveness analysis to compare administering HP-HMB against not administering it, using life years gained (LYG) as the unit of effectiveness and replicating the methodology described by Zhong et al. [19].

The cost-effectiveness analysis was carried out from the perspective of the Spanish National Health System (SNS) using short (90 days post-discharge), medium (180 days, 1 year and 2 years post-discharge), and long-term (5 years post-discharge and lifetime) time horizons. This strategy, assumptions and data included in the analysis were validated by a panel of clinical experts.

### 2.1. Method of Estimating Costs

In estimating the costs, we included the costs resulting from nutritional intervention, from the index hospitalisation, from readmissions and from the use of other healthcare resources within the 90 days post-discharge (including visits to the emergency room, visits to a specialist and to primary care centre/GP, or rehabilitation sessions). All costs were expressed in Euros (year 2016 values).

The costs of the index hospitalisation and of readmissions were estimated based on the costs and mean length of hospital stays registered in the Conjunto Mínimo Básico de Datos (CMBD) [20]. The costs and mean length of stay for the index hospitalisations are shown in Table 1 and Table 2. The costs and mean length of readmissions are shown in Table A1).

In estimating the costs of other healthcare resources, the use of resources was determined based on frequencies observed in the NOURISH study and the unit costs for these resources were taken from the eSalud database [21]. The unit costs and use of resources used for the analysis are shown in Table 1 and Table 3.

For patients treated with HP-HMB, the cost of nutritional intervention was estimated based on the manufacturer’s sale price (PVL) for one dose (220 mL) of Ensure Plus Advance^®^ in Spain (€3.828) and the number of doses per patient observed in the NOURISH Study (94.19 doses/patient) [18]. The patients in the control group were considered to have an intervention cost of €0.

### 2.2. Method of Estimating Effectiveness

To estimate the LYG for the cost-effective analysis with a 90-day time horizon, the survival outcomes observed after the first 90-days post-discharge were taken from the NOURISH study [18].

To estimate the LYG for analyses with time horizons over 90 days, we considered that survival after 90 days did not depend on the intervention received, and the mortality risk level for the general Spanish population was applied, adjusted for sex and age [22].

### 2.3. Incremental Cost-Effectiveness Analysis

The ICER was calculated as the ratio between the costs (total of costs incurred during the index hospitalisation and during the 90-days post-discharge) and the LYG at various time horizons (90 days, 180 days, 12 months, 24 months, 5 years, and lifetime).

### 2.4. Sensitivity Analysis

A sensitivity analysis of the cost-effectiveness assessments was carried out using the 90-day and 180-day time horizons. In both cases, the effect of considering different treatment compliance rates (±10% of the doses observed in the NOURISH study), different lengths of stay in hospital and readmissions (±10% with respect to the values considered in the base case), different use of other resources (±10% with respect to the values of the base case) and the effect of considering only the difference in the cost due to the cost of the HP-HMB treatment (namely, assuming the same use of healthcare resources in both groups), were assessed.

In addition, for the analyses carried out with time horizons of 90 days and 180 days, we estimated the effect on LYG considering the adjustment according to quality of life. In this analysis, the same costs and estimations of LYG as for the incremental cost-effectiveness analysis were considered, and LYG were multiplied by a utility value between 0 and 1 (death and perfect health, respectively) to obtain estimated QALY and to estimate an incremental cost-effectiveness ratio.

To assess the effect of adjusting for LYG according to quality of life in the 90-day time horizon, utilities were estimated using the results of the SF-36 and EQ-5D questionnaires in the NOURISH study [19]. For the estimation based on the SF-36 questionnaire, we used the values observed in the NOURISH study. For the estimation based on the EQ-5D questionnaire, we applied a correction factor to the values observed in the NOURISH study. This factor was based on a comparison between the utility in the general population in the United States and the general population in Spain, and was obtained following the methodology described by Schousboe [23].

In the 180-day time horizon analysis, the same utility value was considered for all patients, regardless of whether they had received HP-HMB or not. A utility value of 0.781, equivalent to the EQ-5D health index for the general population in Spain, in the age range between 75 and 84 years of age, was considered for both groups [24].

## 3. Results

### 3.1. Cost Analysis

In patients who received HP-HMB, the total cost per patient was estimated at €6705.56, and for patients in the control group, the estimated cost was €6372.82 per patient, as detailed in Table 4.

Together, this is an estimated difference of €332.75 per patient between the two groups, in favor of the control group. The costs of intervention and the index hospitalisation were higher for the HP-HMB group than for the control group, whereas, the costs for readmissions and for the use of other healthcare resources in the 90-day post-discharge period were higher in the control group (Table 4). The main causes of readmissions were CHF, COPD and aspiration pneumonia (Table A1).

### 3.2. Effectiveness

In the 90 days post-discharge, the group treated with HP-HMB had a mortality rate of 4.8%, which corresponds to a mean survival of 87.4 days (0.239 LYG), whereas the control group had a mortality rate of 9.7% and a mean survival of 83.9 days (0.230 LYG). The difference between both groups was 0.0096 LYG.

The estimations of LYG with the time horizons of 180 days, 1 year, 2 years, 5 years and lifetime are summarised in Table 5. The differences between both groups varied between 0.024 LYG using the 180-day time horizon and 0.590 LYG using the lifetime time horizon.

### 3.3. Results of the Cost-Effectiveness Analysis with a 90-Day Time Horizon

In the base case analysis with a 90-day time horizon, the incremental cost was €332.75, the incremental benefit was 0.0096 LYG and the ICER was €34,700.62/LYG.

In the sensitivity analysis (Table 6), the ICER varied between €30,941/LYG and €38,461/LYG; these values were obtained by considering, respectively, the use of a lower or higher number of doses than observed in the NOURISH study (±10%). Changes in the length of stay, the use of other resources and the unit costs had reduced impacts on the results of the analysis. When considering the costs of HP-HMB alone, the ICER was €37,600.59/LYG.

In the cost-utility analysis, when the utilities obtained from the SF-36 questionnaire were considered, the estimated gains were 0.140 QALY for patients treated with HP-HMB and 0.129 QALY for patients not treated with this, resulting in a cost-utility ratio of €30,249.61/QALY. However, when we considered the utilities taken from the EQ-5D questionnaire, we obtained a QALY of 0.147 for patients treated and 0.149 QALY for untreated patients in the control arm, and the nutritional intervention was a dominated option compared with the non-intervention.

### 3.4. Analysis of Cost-Effectiveness with Time Horizons of over 90 Days

Considering the incremental cost of €332.75, and considering the differences in LYG observed between both groups with the time horizons of 180 days, 1 year, 2 years, 5 years and lifetime, the respective ICER were €13,711.68, €3377.96, €2253.32, €1127.34 and €563.84/LYG (Table 5).

In the sensitivity analysis of the analysis carried out with the 180-day time horizon, the results varied between €12,225.92/LYG and €15,197.44/LYG (Table 6), whereas, when we only considered the costs of the intervention, the ICER was €14,857.58/LYG. In the cost-utility analysis with the 180-day time horizon, we observed a difference of 0.019 QALY between both groups, and the calculated cost was of €17,557/QALY.

## 4. Discussion

This is the first study that has assessed whether the use of ONS is cost-effective in older adult patients admitted to Spanish hospitals, during hospitalisation and 90-days post-discharge. The results obtained from our study are similar to those observed in a study carried out in the USA based on the results of the NOURISH [19] study and to those observed in other studies assessing the short-term cost-effectiveness of using ONS [25,26].

This study inherits some of the limitations of NOURISH study, such as its limited generalizability due to the inclusion of patients with a limited number of pathologies and its limited follow-up for clinical data (of 90 days). Although the inputs from our study mostly correspond to the data collected as part of a clinical trial carried out in North American hospitals, the profile of the patients included in the NOURISH study are representative of those in an internal medicine unit in Spain [7]; therefore, to a certain extent, the results can be extrapolated to our environment. Not having data available on the use of resources for this type of patient in Spanish hospitals is a limitation. To mitigate this, we used data from the Conjunto Mínimo Básico de Datos (CMBD) to estimate the length of hospital stays (which in all cases were higher than those observed in the NOURISH study) and we validated the use of other healthcare resources (eliminating some of the resources used in the American study, such as admissions to long-stay facilities or to rehabilitation centers). The effects of changing the length of stay and the use of resources were also assessed in the sensitivity analysis, in which modifications in the use of these resources did not have relevant impacts on the results.

As observed in the study published by Zhong et al. [19], the difference in cost between both cohorts rests mainly with the cost of the treatment. Scenarios involving a 10% increase or decrease in the number of doses compared with the base case were the ones with the greatest impact on the results, with differences of ±€3700/LYG compared with the base case using the 90-day time horizon, and ±€1500/LYG using the 180-day time horizon. Given that changes in adherence and compliance with the treatment could affect the validity of the results and the conclusions from this study, it would be appropriate to estimate these parameters in real clinical practice conditions.

This study shows that administering HP-HMB could be cost-effective for malnourished older adult patients admitted to Spanish hospitals, particularly when considering medium and long-term time horizons, which would be the most suitable time horizons for recording the full-scale consequences resulting from this intervention, as recommended by the economic assessment guidelines [27].

Zhong et al. [19] presented the results using two time horizons: 90-days and lifetime. We chose to present the results using different short, medium and long time horizons. This has allowed us to verify that by extending the time horizon by just three months, in addition to the duration of the NOURISH study, the ICER would be €13,711.68/LYG, a figure which would be below €30,000/LYG, a cost-effectiveness threshold commonly accepted in Spain [28]. Using the lifetime horizon, the ICER would be below €600/LYG, a similar result to that obtained by Zhong et al. [19].

Regarding the limitations of our study, in the estimates for medium and long-term survival, the likelihood of death after the 90-day horizon was estimated using mortality rates for the general Spanish population. Not having approximations of mortality rates for a population with features matching those of the clinical trial imposes a restriction. However, considering that the Spanish population aged ≥78 years of age (mean age in the NOURISH study) presents multiple comorbidities [18], and that the majority of fatal events with causes linked to the index hospitalisation would have occurred during the first 90 days post-discharge (and therefore were included in the analysis), we consider this is a reasonable approach.

Another limitation is that we did not have the data that would allow us to estimate the use of resources beyond the 90 days outlined in the NOURISH study. Given that the costs derived from the use of healthcare resources during the 90-days post-discharge (readmissions and other resources) were slightly higher in those patients belonging to the control group than in those in the HP-HMB group, it would be expected that these would continue to be somewhat higher in the control group than in the HP-HMB group, especially in the mid-term. For this reason, to assume that, once past the 90 days, there were no differences in the use of resources between both groups, is a conservative assumption.

It should also be mentioned that the results of the NOURISH study showed uncertainty in assessing quality of life, since dissimilar results were observed when the SF-36 and EQ-5D questionnaires were used. In their base case analysis carried out at 90 days, Zhong et al. [19] calculated a cost–utility ratio considering the results from only the SF-36 questionnaire, as they argued that the EQ-5D would not have sufficient granularity to assess quality of life changes for those patients included in the NOURISH study.

Given the uncertainty regarding quality of life results, in our study, we chose to analyse the cost per LYG in the base case, and to assess the effect of potential adjustments depending on quality of life with both questionnaires in the sensitivity analysis. In the analysis using a 90-day time horizon, applying the utility obtained from the SF-36 questionnaire gave an ICER of around €30,000/QALY, whereas an adjustment based on the results from the EQ-5D resulted in the nutritional intervention being a dominated strategy. In any case, the difference in QALYs for patients receiving and not receiving HP-HMB was minimal, particularly when using the EQ-5D questionnaire (0.147 compared with 0.149). Besides, because of the mortality advantage in patients treated with HP-HMB, the intervention would become cost-effective rather quickly, even when using EQ-5D data. Not having quality of life results beyond 90 days also constitutes a restriction. Assuming that quality of life would be similar in both cohorts, and applying a utility value of 0.781 to the LYG in the analysis carried out using the 180-day time horizon, the intervention was still cost-effective, with an ICER of less than €20,000/QALY. Even considering a scenario where the patient’s quality of life would be very much affected by their baseline comorbidities (for example, assuming a utility value of 0.6), the cost per QALY would still be below €30,000/QALY, with a value of €22,853/QALY.

Despite the high prevalence of malnutrition, its clinical and financial consequences and recommendations for diagnosis and treatment of this condition in malnourished patients [29,30], this is still an underdiagnosed and undertreated condition in Spain. Observational studies carried out in Spain have noted that only 5% of hospital discharges recognise malnutrition as a diagnosis [6], and only 25% of malnourished patients receive any type of ONS [5]. Therefore, it is necessary to implement measures to improve the diagnosis and treatment of malnutrition in specialised units.

In conclusion, the results of this analysis suggest that, for malnourished older adult patients admitted to Spanish hospitals, administering HP-HMB during hospitalisation and in the 90 days post-discharge could be a cost-effective intervention that would improve survival with a reduced marginal cost. These results should be confirmed in observational longitudinal studies.

## Figures and Tables

**Table 1:** Unit Costs.

Healthcare Resources	Cost	Source
**Index hospitalisation**		
Chronic heart failure (cost/day)	€488.39	Conjunto Mínimo Básico de Datos (CMBD) [20]
Acute myocardial infarction (cost/day)	€941.50	
Pneumonia (cost/day)	€536.63	
Chronic obstructive pulmonary disease (COPD) (cost/day)	€627.76	
**Other healthcare resources**		
Visits to hospital emergency department (cost/visit)	€168.96	eSalud database [21]
Visits to specialist (cost/visit)	€75.90	
Visits to primary care (cost/visit)	€36.08	
Rehabilitation sessions (cost/session)	€21.65	

**Table 2:** Cost of index hospitalisation.

Diagnosis	Control			Beta-hydroxy-beta-methylbutyrate (HP-HMB)		
	% Patients	Length of Stay (Mean, Days)	Cost/Patient	% Patients	Length of Stay (Mean, Days)	Cost/Patient
Chronic heart failure	25.2%	8.47	€1044.20	25.2%	8.47	€1044.07
Acute myocardial infarction	8.1%	7.1	€540.83	9.6%	7.1	€640.70
Pneumonia	32.4%	8.04	€1396.27	30.4%	8.04	€1309.51
COPD	34.3%	7.52	€1619.42	34.8%	7.52	€1643.97

**Table 3:** Cost of using other healthcare resources.

Healthcare Resources	Control			Beta-hydroxy-beta-methylbutyrate (HP-HMB)		
	% Patients	No of Visits per Patient (Median, N)	Cost/Patient	% Patients	No of Visits per Patient (Median, N)	Cost/Patient
Visits to Emergency Room	21%	1.00	€35.48	19%	1.00	€32.10
Visits to specialist	45%	3.00	€102.47	48%	2.00	€72.86
Visits to primary care facility	56%	2.00	€40.41	58%	2.00	€41.85
Rehabilitation sessions	11%	12.00	€28.57	12%	12.00	€31.17

**Table 4:** Total cost per patient over study period.

Healthcare Resources	Control	HP-HMB	Difference
Nutritional intervention	€0	€360.55	€360.55
Index hospitalisation	€4600.71	€4638.24	€37.53
Readmissions	€1565.17	€1528.77	−€36.40
Other healthcare resources	€206.93	€177.99	−€28.94
Total	€6372.82	€6705.56	€332.75

HP-HMB: Beta-hydroxy-beta-methylbutyrate.

**Table 5:** Life years gained (LYG) and Incremental Cost-Effectiveness Ratio (ICER) at various time horizons.

Time Horizon	Control (LYG)	HP-HMB (LYG)	Difference in LYG	ICER (€/LYG)
90 days	0.23	0.24	0.010	34,700.62
180 days	0.49	0.52	0.024	13,711.68
1 year	1.79	1.89	0.099	3377.96
2 years	2.59	2.74	0.148	2253.32
5 years	4.71	5.01	0.295	1127.34
Lifetime	6.82	7.41	0.590	563.84

**Table 6:** Sensitivity analysis of the Incremental Cost-Effectiveness Ratio (ICER) with time horizons of 90 and 180 days.

Analysis		ICER 90-Day Time Horizon (€/LYG)	ICER 180-Day Time Horizon (€/LYG)
Base case		34,700.62	13,711.68
HP-HMB dose price	−10%	30,941.00	12,225.92
	+10%	38,461.00	15,197.44
Length of in-hospital stay	−10%	34,806.82	13,753.64
	+10%	34,830.42	13,762.97
Use of other resources	−10%	31,984.46	12,638.41
	+10%	31,380.86	12,399.90
Unit costs	−10%	32,090.65	12,680.37
	+10%	31,510.66	12,451.19
Cost of treatment alone		37,600.59	14,857.58

## Appendix A

**Table A1:** Readmissions: cost per day, mean length of stay and number of hospitalisations per treatment group.

Diagnosis	Cost/Day	Mean Length of Stay (Days)	No of Hospitalisations During 90-Day Post-Discharge Period	No of Hospitalisations During 90-Day Post-Discharge Period
			Control (*n* = 309)	HP-HMB (*n* = 313)
Abdominal pain	€587.24	4.39	0	1
Acute exacerbation of COPD	€549.20	7.73	0	1
Acute febrile illness (caused by a urinary tract infection)	€496.47	7.05	1	0
Acute myocardial infarction	€941.50	7.10	1	4
Acute or chronic diastolic dysfunction in the context of severe mitral stenosis	€1097.31	12.75	0	1
Acute respiratory failure	€639.26	8.10	1	0
Altered mental status	€405.25	8.81	1	0
Anaemia	€591.51	6.58	1	0
Microcytic anaemia	€591.51	6.58	0	1
Angina pectoris	€650.88	5.02	1	0
Aortic aneurysm	€1263.44	10.50	0	1
Aortic valve stenosis	€1332.33	10.92	0	1
Weakness	€632.27	5.62	1	0
Asthma	€528.45	5.95	1	0
Atrial fibrillation	€731.93	5.12	2	1
Back pain	€640.41	5.86	0	2
Bradycardia	€937.02	4.48	0	2
Elevated brain natriuretic peptide	€806.59	4.06	1	0
Cardiac arrest	€940.86	11.59	2	0
Congestive heart failure	€488.39	8.47	12	9
Cerebrovascular accident	€610.09	9.13	3	2
Chronic heart failure	€488.39	8.47	1	1
Exacerbation of chronic heart failure	€488.39	8.47	0	1
Chronic obstructive pulmonary disease	€627.76	7.52	1	0
Chronic obstructive pulmonary disease	€627.76	7.52	21	21
Chronic obstructive pulmonary disease with acute or chronic respiratory failure	€549.20	7.73	0	1
Colitis	€582.83	4.76	0	1
Ischaemic colitis	€499.69	9.22	1	0
Confused state	€647.03	14.16	2	0
Chronic heart failure	€488.39	8.47	1	0
Exacerbation of chronic heart failure	€488.39	8.47	0	1
Exacerbation of COPD	€549.20	7.73	0	1
Deep vein thrombosis	€607.28	6.15	1	0
Dehydration	€598.23	6.39	0	1
Malfunction of device	€797.58	6.82	2	0
Diarrhoea	€522.97	6.87	0	1
Diverticulitis	€585.56	7.85	0	2
Dizziness	€563.90	4.15	0	1
Shortness of breath	€752.27	4.54	3	3
Insufficient weight gain	€519.37	5.23	0	1
Fall	€368.67	10.40	2	4
Fracture	€368.67	10.40	0	1
Gastrointestinal bleeding	€578.93	6.87	1	2
Groin pain	€559.42	7.14	1	0
Haematuria	€378.61	6.36	0	2
Haemoptysis	€528.26	7.07	0	1
Exacerbation of heart failure and COPD	€488.39	8.47	0	1
Hip fracture	€827.82	10.51	3	1
Fracture to the humerus	€845.80	9.28	1	0
Hyperkalemia	€450.78	8.92	0	1
Hypophagia	€444.36	7.73	1	0
Hypoxia	€561.70	6.62	1	0
Obstructed inguinal hernia	€1216.73	1.98	0	1
Bowel obstruction	€559.55	7.60	0	1
Malnutrition	€375.37	11.25	1	0
Changes in mental status	€405.25	8.81	1	2
Multiple organ failure	€649.56	11.75	1	0
Chest pain-non-cardiac	€544.39	4.09	0	1
Oedema	€577.94	6.74	0	1
Pancytopenia	€449.84	9.00	1	0
Pleurisy	€514.77	9.45	0	1
Pneumonia	€536.63	8.04	0	1
Aspiration pneumonia	€601.15	9.24	7	10
Aspiration pneumonia with respiratory failure	€536.63	8.04	2	0
Pneumothorax	€706.72	6.11	1	0
Presyncope (feeling faint)	€572.49	5.39	0	1
Pulmonary embolism	€517.40	9.18	1	0
Acute renal failure	€505.71	8.99	1	1
Breathing difficulties	€752.27	4.54	1	0
Respiratory failure	€639.26	8.10	3	0
Sepsis	€597.94	10.52	0	1
Septic shock	€624.52	14.22	0	1
Septic shock caused by staphylococcus	€605.04	14.91	1	0
Syncope	€572.49	5.39	0	2
Systemic inflammatory response syndrome	€678.84	10.05	1	0
Toxicity from various agents	€689.30	5.85	0	1
Transient ischaemic attack	€573.24	5.25	0	1
Elevated troponin	€806.59	4.06	1	0
Uncontrolled hypertension	€626.18	5.28	0	1
Urinary tract infection	€496.47	7.05	1	0
Vertigo	€563.90	4.15	1	0
Worsening of COPD and respiratory failure	€639.26	8.10	1	0
